# Multi-country review of ITN routine distribution data: are ANC and EPI channels achieving their potential?

**DOI:** 10.1186/s12936-022-04373-6

**Published:** 2022-12-03

**Authors:** Jane E. Miller, Kezia Malm, Aimain Alexis Serge, Marcellin Joel Ateba, Peter Gitanya, Doudou Sene, Emmanuel H. Kooma, Balla Kandeh, Lilia Gerberg, Luigi Nuñez

**Affiliations:** 1 PMI VectorLink, PSI, Washington, DC USA; 2 National Malaria Control Programme, Accra, Ghana; 3 National Malaria Control Programme, Abidjan, Côte d’Ivoire; 4 National Malaria Control Programme, Yaoundé, Cameroon; 5 National Malaria Control Programme, Dodoma, Tanzania; 6 National Malaria Control Programme, Dakar, Senegal; 7 National Malaria Elimination Programme, Lusaka, Zambia; 8 National Malaria Control Programme, Banjul, The Gambia; 9 U.S. President’s Malaria Initiative, USAID, Washington, DC USA

**Keywords:** Routine, Continuous distribution, ANC, EPI, ITNs, Pregnant women, Infants

## Abstract

**Background:**

Routine continuous distribution (CD) of insecticide-treated nets (ITNs) has been an important part of an overall ITN strategy to complement mass campaigns since the early 2000s. The backbone of CD implementation for many sub-Saharan African countries is distribution through antenatal care (ANC) and Expanded Programme for Immunizations (EPI) channels. Performance of these channels is often not monitored closely at the national level, nor is it reviewed globally, unlike the oversight provided to mass campaigns. The question as to why every eligible pregnant woman and child attending these services does not get an ITN remains important and yet, unanswered.

**Methods:**

ANC and EPI issuing rates from seven countries were reviewed with the aim of conducting a blinded multi-country analysis. Monthly data from January to December 2021 was extracted from each country’s health management information system and analysed jointly with a National Malaria Control Programme (NMCP) focal point. VectorLink CD assessment reports were also reviewed to glean key findings.

**Results:**

ITN issuing rates varied across countries at ANC (31% to 93%) and EPI (39% to 92%). Across the seven countries, the median ITN issuing rate was 64% at ANC and 78% at EPI. Results varied greatly across months per country at both ANC and EPI. NMCP focal points are aware that mass campaigns often negatively affect implementation of ITN distribution through ANC and EPI, even though global and national guidelines emphasize sustaining CD during campaigns. Concerns were also raised about the standard ITN issuing rate indicator at ANC and even more so at EPI due to the denominator.

Findings from CD assessments were similar across countries: ITN stock was inconsistent and sometimes inadequate, and updated guidelines on ITN distribution and utilization and funding for social behaviour change activities were lacking at the facility level.

**Conclusion:**

The importance of optimizing ANC and EPI routine channels cannot be underscored enough. They are at the frontline to protect the most biologically vulnerable populations, i.e., pregnant women and unborn and young children. Although there are encouraging signs of improvement in issuing rates with some countries reaching optimal rates, further improvements are needed to ensure that every pregnant woman and young child receives the ITN to which they are entitled.

**Supplementary Information:**

The online version contains supplementary material available at 10.1186/s12936-022-04373-6.

## Background

The World Health Organization (WHO) World Malaria Report (WMR) 2021, estimates that globally 1.7 billion malaria cases and 10.6 million malaria deaths were averted by all malaria control efforts, in the period 2000–2020. Most of these cases (82%) and deaths (95%) averted were in the WHO African Region [1]. It is estimated that from 2000–2015, 68% of the 663 million cases averted was due to ITNs [2]. Despite these impressive gains, the global malaria community is concerned that these results are plateauing, as reductions in the rate of malaria cases have dramatically declined. Globally, there were an estimated 241 million malaria cases in 2020 in 85 malaria endemic countries, an increase from an estimated 227 million in 2019. The majority of this increase came from countries in the WHO African Region [1].

In 2020, in 33 moderate and high transmission countries in the WHO African Region, there were an estimated 33.8 million pregnancies, of which 11.6 million (34%) were exposed to malaria infection during pregnancy and it is estimated that this resulted in 819,000 children with low birthweight in these countries. Given that low birthweight is a strong risk factor for neonatal and childhood mortality, averting a substantial number of children with low birth weight will save many lives [1]. Although malaria deaths in children under five years old (“child”) dropped in 2018, this age group still accounted for two-thirds (67%) of all malaria deaths worldwide [3].

In sub-Saharan Africa, the percentage of the population sleeping under an ITN increased considerably between 2000 and 2020, for the entire population (from 2 to 43%), for children aged under five years (from 3 to 49%), and for pregnant women (from 3 to 49%) [1].

Global efforts to reduce the burden of malaria include large-scale distribution of ITNs through mass campaigns and CD channels. CD channels include distribution of nets to pregnant women in ANC clinics and to young children during EPI visits, as well as schools, community-based distribution, and the private sector which includes social marketing.

In order to provide protection for those most biologically vulnerable to malaria, pregnant women and young children, distribution of ITNs via routine health services began more than 20 years ago with the first documented pilots in Tanzania—the Ifakara and Swiss Tropical and Public Health Institute Kilombero Net (KINET) project in 1996 and the Population Services International (PSI) Social Marketing of ITNs (SMITN) project in 1998. Through KINET, nets and net treatment were sold in the private sector. Pregnant women at ANC clinics were given a discount voucher which reduced the price of the net by about 20%. The SMITN Project’s Lea Mwana nets were sold to pregnant women for $0.50 at the clinics, the first such initiative recorded that had nets available at clinics. The payments were used by the clinics for maintenance and fuel costs [4]. Both projects showed promising results for implementation feasibility and increasing ITN ownership among pregnant women and the success of these pilots led to a nationwide voucher scheme called Hati Punguzo or the Tanzania National Voucher Scheme [5]. Building on these experiences, Malawi implemented the first nationwide ANC ITN delivery programme in 2002, with household coverage with at least one ITN increasing from 27.4% in 2004 [6] to 56.8% in 2010 [7], with 85% of all nets distributed through continuous channels [8]. These impressive results were obtained before the first mass campaign in Malawi (2012).

For well over a decade, the WHO has produced recommendations and guidelines, supported by Roll Back Malaria Partnership to End Malaria (RBM), emphasising the CD of ITNs for free through ANC and EPI, to complement mass campaigns and improve coverage. In 2011, the RBM Vector Control Working Group recommended giving higher priority to routine services, such as ANC and EPI, as a means of ITN distribution to sustain universal coverage of ITNs and further stated that CD channels should be functional before, during, and after the mass distribution campaigns to avoid any gap in universal coverage of ITNs [9].

A 2012 analysis found that supplementing a universal mass campaign with ANC delivery in medium to high transmission settings would achieve a 1.4 times higher mortality reduction than campaign delivery alone, reflecting that those children born in the years between campaigns would otherwise have access to old nets or no nets at an age of high risk. The relative advantage of supplementary ANC delivery was still present though smaller if malaria transmission levels were lower or if there was a strong mass effect achieved by mass campaigns [10].

Health facility CD channels were found to be more cost-effective than mass campaigns for averting Disability Adjusted Life Years, deaths, and cases of malaria [11]. A review of four types of continuous delivery systems (ANC, EPI, schools, and community/health facility) in six countries showed that these strategies can continue to deliver nets at a comparable cost to mass distributions, especially from the perspective of the donor [12]. Secondary analysis of national survey data by Theiss-Nyland et al. [13] found that an average of 54% of children slept under an ITN in countries with both ANC and EPI distribution compared with 34.3% for ANC only and 24.7% with no facility-based distribution. Each additional ITN distribution policy increased average net use among children under five by 13%. There are obvious advantages to having both channels operating efficiently.

Thus, many countries have considerable experience implementing some form of routine ITN distribution. Routine CD of ITNs has been seen as an important part of an overall ITN strategy since the early 2000’s and the backbone of CD implementation for many sub-Saharan countries has been through ANC and more recently, EPI channels but not necessarily both. According to the WHO WMR 2021, in Africa, 32 countries were distributing ITNs through ANC and 24 through EPI clinics in 2020 [1]. In 2019, 80% of pregnant women in sub-Saharan Africa used ANC services at least once during their pregnancy [2]. The percentage of the population sleeping under an ITN increased considerably between 2000 and 2020, but since 2017, indicators for ITN access and use in sub-Saharan Africa have been declining [3].

Despite the years of collective and country-individual experience, most CD programmes still struggle to maintain a high-quality service. This can be seen in frequent stockouts at health facilities and other issues. A few studies have highlighted issues such as adherence to national issuing guidance regarding eligibility, recording issuing (in register and on health card), and lack of interpersonal communication when issuing in the performance of ANC and EPI channels. A four-country qualitative review of the distribution of ITNs through health facilities found that it was more effectively integrated through ANC than through EPI because of (a) stronger linkages and involvement between malaria and reproductive health programmes, as compared to malaria and EPI, and (b) more complete programme monitoring for ANC-based distribution, compared to EPI-based distribution. The authors concluded that there are opportunities for improving the distribution of ITNs through these channels, especially in the case of EPI. They also argue that, for both ANC and EPI, integrated distribution of ITNs has the potential to act as an incentive, improving the already strong coverage of both essential services [14].

Another study highlighted that the routine distribution channels appear to be under-utilized, especially EPI-based distribution. Out of 38 country programmes reporting on ITN distribution, data to calculate ITN availability through ANC and EPI were only found in 17 and 16 countries, respectively. The availability ratios were based on the number of ITNs reportedly distributed by national ANC programmes over a three-year period through 2012 divided by the number of women reportedly attending ANC. Over a three-year period through 2012, distribution through ANC accounted for 9% of ITNs distributed, and ITNs distributed through EPI accounted for 4%. The ITN availability ratios achieved were 55% through ANC and 34% through EPI. The authors conclude that quality data from more countries are needed for consistent and reliable programme performance monitoring and that a greater focus on routine data collection, monitoring, and reporting on ITNs distributed through both ANC and EPI can provide insight into both strengths and weaknesses of CD and improve the effectiveness of these delivery channels [15].

The performance of these health facility channels is rarely monitored closely at the national level, nor is it reviewed at the global level, unlike the lens brought to mass campaigns. Although many of the reasons as to why all the most biologically vulnerable—pregnant women, infants, and young children attending ANC and EPI—do not get an ITN are known, there is still a long way to go until every pregnant woman and young child in sub-Saharan Africa receives the ITN to which they are entitled.

## Main text

The purpose of this activity was to further understand the challenges and, where necessary, identify the barriers that remain to optimal distribution through these channels, by reviewing the routine programme data from routine continuous distribution channels from several countries. In each country, discussions were held with the NMCP Director and other key staff about data collection and review with the aim of writing together an anonymous multi-country paper. The countries were from East, Central, and West Africa.

To achieve this, monthly ANC and EPI data for the eligible populations and ITNs issued per channel from January to December 2021 was requested and obtained from the NMCPs of seven countries. This time period was selected as it was the most up-to-date annual data available. Other countries were approached through the United States President’s Malaria Initiative (PMI) VectorLink team, but the data had not yet been entered into the national health management information system (HMIS) or were unavailable for other reasons.

Secondly, for a better understanding and interpretation of these data, discussion sessions and email exchanges were conducted with focal points designated by the NMCP.

Thirdly, six ITN continuous distribution assessments (CDAs) carried out with the support of the PMI VectorLink project were reviewed to identify key findings and recommendations across the countries. These included four of the seven countries from which ANC/EPI data was obtained. Based on the frequency of citation from the assessments, recommendations that would strengthen the routine CD process and maintain optimal ITN coverage levels through routine CD channels were listed.

### HMIS data collection

#### Data Collection from NMCPs

In all countries, health facilities collect routine data as part of service delivery using some mix of registers (typically distinct for ANC and EPI), tally sheets, and stock inventory control cards. The health facilities then collate the data from these source documents into monthly standardized reporting forms developed and maintained by the government. Data from these forms are then entered into the national HMIS. For all countries included in this activity, every HMIS is based on District Health Information Software (DHIS2), an open-source software platform for reporting, analysis, and dissemination of data used in more than 73 countries around the world. Typically, facilities submit paper-based copies of the monthly reporting form to their health district team who then enters the data into the national HMIS. In some cases, facilities may directly enter their monthly health data into the national HMIS themselves. The timing and frequency of data entry and data quality activities differ by country.

Data used for this activity were extracted from each country’s HMIS in the first quarter of 2022. To do this, an Excel file was prepared to capture the values for the number of pregnant women visiting health facilities for ANC, the number of nets given to pregnant women at ANC, the number of children under five years of age attending for vaccination, and the number of nets given to the children.

The Excel file was designed to record data down to the second level of aggregation, corresponding to the district. This file was then shared with the NMCPs of the countries of interest with all the necessary instructions on how to copy the data from the DHIS2 and paste it in. Countries were asked to provide data from January to December 2021. If a country did not distribute ITNs through one channel or did not provide a month of data, it was reported as N/A.

The influence of the COVID-19 pandemic on data collection and reporting was not directly investigated and nor was the issue of COVID-19 mentioned in any of the NMCP discussions.

#### Data analysis by PMI VectorLink

The data received from the NMCPs were quality-controlled, and abnormalities such as the number of nets issued being much higher than the number of pregnant women or children received were identified and raised to the respective government for their awareness. However, given the nuances in facility based ITN indicators (explained more in the conclusion), those data points were kept in the analyses. A detailed analysis was then carried out to calculate the rates of nets issued to each group of individuals at national, regional, and district levels. This allowed spatial and temporal trends to be plotted for the 2021 calendar year.

The performance of CD of ITNs through health facilities is measured through the issuing rate at ANC and EPI. The issuing rate at ANC is calculated as the number of pregnant women who received an ITN at ANC divided by the number of pregnant women at their first ANC visit times 100%.

$${Issuing\,rate}_{ANC}=\frac{\# of\, pregnant \,women\, who\, received \,an\, ITN \,at\, ANC}{\# of\, pregnant \,women\, at\, first\, ANC \,visit} \times 100\%$$

The issuing rate at EPI is calculated as the number of children who received an ITN at EPI divided by the number of children receiving their second dose of the measles-rubella (MR) vaccine times 100%.

$${Issuing\, rate}_{EPI}=\frac{\# of \,children\, who \,received \,an \,ITN \,at \,EPI}{\# of \,children\, receiving \,MR2\, vaccine \,dose\, at \,EPI} \times 100\%$$

These four elements are reported by health facilities as part of monthly routine reporting of health services. As previously mentioned, these data are entered into the national HMIS which can then be analysed through live dashboards or exported for further analyses in other software such as Excel or STATA. For this activity, Excel was used for the descriptive analyses, including temporal graphs and tables. In addition, spatial analyses were conducted using ArcGIS Desktop 10.8.2 but the visuals are not included to respect the anonymity of countries.

The 2021 ITN issuing rates at ANC and EPI per country as well as the grand total (i.e., summing the numerators and denominators across all countries before division) were calculated, as well as the average and median results using the national results (seven for ANC, six for EPI). No adjustments were made for different routine data quality across the countries.

#### Data review with NMCP teams

To better interpret the data from each country and draw appropriate conclusions, discussion sessions were held with the focal point identified by the NMCPs. Dependent on the results, the format of each discussion session differed. For example, some were a one-hour virtual call to present and discuss the results, facilitated by guiding questions, and others were a series of email threads with a virtual call to focus more on the challenges. The open discussions were centred on the issues perceived to be key to the functioning of the routine distribution.

### Review of CDAs

The PMI VectorLink CDA [16] is a comprehensive qualitative assessment of continuous ITN distribution channels which provides NMCPs and key health partners with information to achieve their ITN coverage goals, informed by global best practices. The assessment is conducted as a review of existing distribution systems in place, as it focuses on document review and key informant interviews with health personnel and community partners involved in ITN distribution.

Two primary questions guide the assessment: To what extent is continuous ITN distribution implemented according to existing international and national guidelines and plans across all levels and components (e.g., coordination, beneficiary registration, quantification, personnel, etc.)? And, secondly, among each component, what improvements can be identified to deliver efficiency gains in continuous ITN distribution in support of ITN access?

CDAs have been carried out in six countries from 2018–2022 (Niger, Senegal, Cameroon, Burkina Faso, Cote d’Ivoire, and Zambia). To conduct the assessment, NMCPs and the assessment implementing partners used qualitative methods, including record and document review, and a series of semi-structured interviews with national, sub-national, and facility-based stakeholders were carried out with support of a discussion guide. The sample discussion guide was developed with the Ministry of Health (MOH) and ITN stakeholders. The notes from the semi-structured interviews were reviewed and compiled into the CDA country report.

The reports of all six CDAs were reviewed by the PMI VectorLink team, and key findings and recommendations were noted and used to inform the NMCP discussions.

## Results

### Analysis of routine HMIS data

**Fig. 1:**
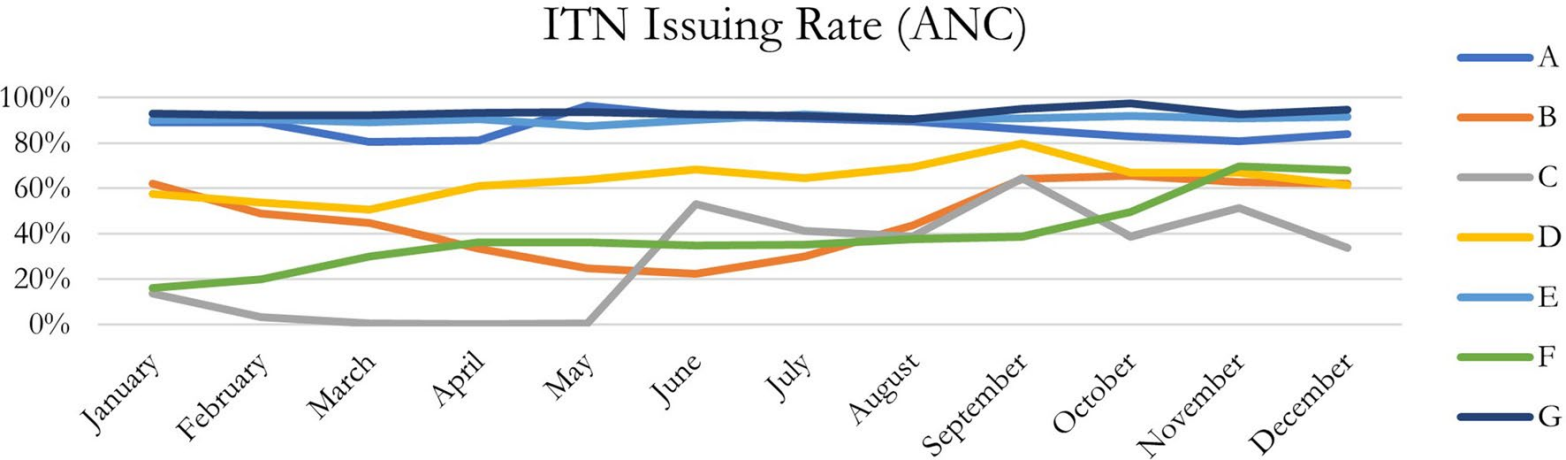
Monthly ITN issuing rate at ANC, per country, 2021

**Fig. 2:**
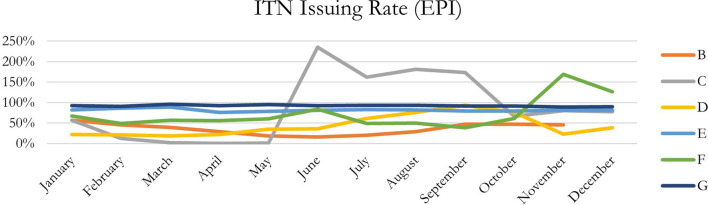
Monthly ITN issuing rate at EPI, per country, 2021

Seven countries provided ANC data and six of the seven countries provided EPI data for the period January–December 2021. ITN issuing rates for 2021 varied considerably across countries (31% to 93% for ANC, and 39% to 92% for EPI). Using data from all countries, the total ITN issuing rate at ANC and EPI were 75% and 71%, respectively. The averages using national results were 65% (ANC) and 69% (EPI), and the medians using national results were 64% (ANC) and 78% (EPI). From a descriptive analysis of the monthly data at ANC (Fig. 1) and EPI (Fig. 2), performance tended to improve about halfway through the year and remained higher during the second half of the year than the first half of the year in each respective country. This may have been due to the rainy season causing increased focus and prioritization by facility and government actors, in addition to potentially more demand by community members. The timing of ITN distribution from the central level to the health facilities may also have an effect; some countries distribute quarterly, some bi-annually and some even annually. The timing of donor funding and procurement may also have had an impact on distribution.

In addition, spatial analyses were carried out to highlight if there were patterns particularly at border areas to see if two countries sharing a border had similar performance at those border areas. No similarities of issuing rate performances at borders were found.

### Data review with NMCPs

Key findings from the data review with NMCPs included the following:

It was highlighted that it is likely mass ITN campaigns explain some of the monthly variance for distribution at health facilities. Although global and national guidance dictate that focus on CD should remain constant even if a campaign is being planned or is underway. In reality, CD channels tend to be deprioritized during mass campaigns. This includes even at the facility and community level, as health facilities and health workers are sometimes diverted to campaign activities and community health workers may support social behaviour change (SBC) and communication of campaign registration and distribution dates. Nets may sometimes also be reallocated from the facilities to the mass campaign. During 2021 three out of the seven countries had some sort of mass campaign and two of these campaigns seemed to effect issuing rates.

Also, there was some concern about the ANC issuing rate indicator. Though the indicator is mostly accurate and precise in measuring true ANC ITN issuance, the denominator is still a proxy (i.e., using first ANC visit). It is known anecdotally and there is growing evidence that many women receive a net at a subsequent visit, perhaps due to a stockout at the facility during the first visit. This is allowed per national guidelines, to help ensure that every woman receives a net during their pregnancy. However, it is found then that some facilities have results over 100% (even above 200%) which may in fact be accurate. This, in turn, has implications for routine monitoring and programmatic decision-making.

There was even more concern for the EPI issuing rate indicator. Increasingly, countries are not only issuing nets to children at EPI; national guidelines may allow nets to be given at outpatient visits (if the child is not documented to have received a net already in their child booklet). The most customary practice for the denominator is to use the number of second MR vaccines administered as that visit typically lines up with when a healthcare provider should issue an ITN to a child at EPI. However, stockouts of the MR vaccine can cause the number of administrations to be substantially lower than the number of nets issued, so the performance indicator may seem to perform well for a facility when in fact the high result is due to an inaccurate (and low) denominator. In addition, in many countries, high vaccine coverage is due to mass vaccination campaigns, not routine services at health facilities, so the true coverage of children receiving a net is much lower given that there are many children who may not attend EPI since they received their critical vaccinations and Vitamin A supplements through campaigns.

### Key findings and recommendations from CDA

Table 3 highlights the eight components of ITN distribution which are reviewed during a CDA, with the key findings and key recommendations common to the six countries.Additional file 1: Table S1. Components of ITN CD and key findings and recommendations from PMI VectorLink CDAs.

These findings are consistent with some of the NMCP discussion feedback and are very much aligned with findings from other such initiatives [18–20]. All the countries reviewed had mechanisms in place for routine distribution with high level MOH support. However, it was found that there was less coordination across ministry departments.

Three key findings are:

1. All countries faced **inconsistent and sometimes inadequate ITN supplies** and sometimes across the entire supply chain. At the national level, this was usually a result of the fact that procurement may be entirely dependent upon donor support. This can lead to stockouts when donor support is not timely or is not in line with programme requirements. There was often a lack of resources to support last-mile distribution at district level (including distribution to hard-to-reach areas). The use of Central Statistics Office population data in commodity quantification instead of target community head counts compounded this challenge and led to prolonged stockouts at service delivery points. These factors have likely contributed to low ITN issuance rates nationally. A key recommendation is to consider, or to increase, domestic financing for ITNs to ensure that these channels do not face these issues.
2. **Lack of updated guidelines** and training or orientation **on the distribution and utilization of ITNs.** Guidelines were either old and out of date or simply not available. Additionally, it was found that orientation of staff on the programme sometimes was not taking place at any level over the years, despite the (outdated) guidelines found in one country that did stipulate training/orientation as a requirement every two years. The combination of these two factors has led to a lack of clear understanding among some programme implementers as to who the end users are for the ANC and EPI channels at the health facility level. This has occasionally resulted in ITNs being issued to end users who are outside of the categories stipulated by the guidelines or nets have not been given to those who are eligible.
3. **Budgetary support for SBC activities.** Working collaboratively with existing community structures, health facility staff were often found to provide information on malaria prevention and ITN use and care, but in most countries, neither the provinces, districts, nor health facilities have specific budgetary support for this. Because of the lack of budgetary support, SBC activities pertaining to ITNs are mostly limited to health talks during ANC and EPI sessions, which are usually carried out on different days at separate times and to non-ITN-specific community activities on which the ITN programme can piggyback. Information, Education, and Communication materials are generally also unavailable at provincial and lower levels and, when available, are mostly in English or French. This renders the materials non-user friendly to local target audiences that are unable to read and understand these languages. The current situation has the obvious potential to limit the extent to which behaviours such as ITN misuse (which several facility and district staff reported was common) and inconsistent and incorrect ITN use are addressed.

## Conclusion

### Variable results

The results show mixed results regarding optimal distribution across ANC and EPI channels, countries, and within these countries across regions and seasons. Table 1 shows these findings.

**Table 1:** ITN issuing rate at ANC and EPI per country, 2021

Country	ITN issuing rate (ANC)	ITN issuing rate (EPI)
A	87%	N/A
B	48%	39%
C	31%	88%
D	64%	41%
E	91%	81%
F	40%	75%
G	93%	92%
Grand total	**75%**	**71%**
Average (national)	**65%**	**69%**
Median (national)	**64%**	**78%**

Totals highlighted in bold.

2021 results across the seven countries demonstrate median performance of CD of ITNs through health facilities at 64% for ANC and 78% for EPI. Results vary across countries (31% to 93% for ANC, and 39% to 92% for EPI).

Furthermore, how one understands the current state of performance drastically changes based on which aggregation method you use. For example, the averages using national results were 65% (ANC) and 69% (EPI), the medians were 64% (ANC) and 78% (EPI), and the grand totals across all seven countries were 75% (ANC) and 71% (EPI). This means that global performance (in this case, using six countries for EPI and seven countries for ANC) may be as low as 69% or as high as 78% for EPI and as low as 64% or as high as 75% for ANC. A single aggregation method is not endorsed and instead the importance of looking at performance using these different methods to arrive at a more comprehensive understanding of the lower and upper bounds of where actual performance lies is recommended.

In addition, through descriptive analyses of the monthly data at ANC and EPI, with several countries, it seems performance tends to increase around halfway through the year and maintains higher performance during the second half of the year in each respective country. This may be due to the rainy season causing increased focus and prioritization by facility and government actors, in addition to potentially more demand by community members (Figs. 1 and 2, Tables 2 and 3). There may also be an affect due to mass campaigns depending on when and how they were carried out and the frequency of the distribution of routine ITNs from the central levels.

**Table 2:** Monthly ITN issuing rate at ANC, per country, 2021

ITN issuing rate (ANC)	Country							
Month	A	B	C	D	E	F	G	Grand Total
January	89%	62%	14%	57%	90%	16%	93%	74%
February	89%	49%	3%	54%	90%	20%	92%	72%
March	81%	45%	0%	51%	89%	30%	92%	70%
April	81%	33%	0%	61%	91%	36%	93%	72%
May	96%	25%	0%	64%	87%	36%	94%	72%
June	92%	22%	53%	68%	90%	35%	93%	72%
July	91%	30%	41%	64%	93%	35%	92%	74%
August	90%	43%	39%	69%	91%	38%	91%	75%
September	86%	64%	64%	80%	91%	39%	95%	79%
October	83%	66%	39%	67%	92%	49%	97%	80%
November	81%	63%	51%	67%	91%	70%	93%	81%
December	84%	62%	34%	61%	92%	68%	95%	81%
Grand total	87%	48%	31%	64%	91%	40%	93%	75%

**Table 3:** Monthly ITN issuing rate at EPI, per country, 2021

ITN issuing rate (EPI)	Country							
Month	A	B	C	D	E	F	G	Grand Total
January	N/A	57%	57%	22%	82%	67%	92%	71%
February	N/A	46%	13%	21%	86%	49%	91%	67%
March	N/A	39%	2%	18%	89%	56%	96%	67%
April	N/A	29%	0%	22%	76%	56%	92%	65%
May	N/A	18%	2%	35%	79%	61%	95%	65%
June	N/A	16%	235%	36%	81%	84%	92%	68%
July	N/A	20%	162%	61%	83%	49%	93%	66%
August	N/A	29%	181%	76%	82%	50%	93%	69%
September	N/A	47%	174%	94%	80%	39%	92%	72%
October	N/A	47%	66%	76%	79%	61%	92%	73%
November	N/A	46%	80%	23%	81%	169%	89%	81%
December	N/A	N/A	77%	39%	81%	126%	90%	94%
Grand total	N/A	39%	88%	41%	81%	75%	92%	71%

Interestingly, COVID-19 was not raised as an issue during any discussions.

The purpose of this review was to explore whether ANC and EPI channels are reaching their potential in ensuring that all pregnant women receive nets to protect both themselves and their unborn and/or young children. ITN delivery through ANC clinics is both a vector control and malaria in pregnancy initiative. In addition, whether the EPI channel is as effective as it could be in reaching young children who may no longer be protected by the original net if it is worn out or is now being used by the newly pregnant mother without her previous child or simply that these children’s household may not have received a net or enough nets to cover everyone in the household was investigated.

As the 2019 Theiss-Nyland review [12] reports, routine distribution programmes, when implemented together, greatly improve net ownership and use and provide nets to vulnerable children who may not otherwise be covered. The reason for improvement is that a net given to a pregnant woman will be near to the end of its useful life by the time the infant is a toddler. A second facility based distributed ITN, via EPI, beyond the one given at ANC, has the potential to increase the total ITNs in a household, increasing the number and proportion of homes with universal access and improving ITN use in children under five. They conclude by saying that beyond merely “keeping-up” ITN coverage, the combination of these services can improve coverage, making them a valuable tool for the control and elimination of malaria.

### Consistent reasons for issuing variability

From the NMCP discussions and the CDAs, several key challenges were found including lack of stock, guidance tool, training opportunities, and the lack of SBC funding. One of the key issues facing health facilities is stockout due to challenges with getting the nets to the facilities. District health management teams often have limited vehicles and fuel supplies, and nets are a bulky commodity. To address this issue, countries such as Ghana are implementing Last Mile Delivery (LMD) which is a commodity supply mechanism that ensures scheduled supply from the regional medical stores to health facilities. LMD shifts the burden of health commodity supply from district health teams to the regional medical stores, which are better equipped with storage facilities and transport. This initiative is being rolled out nationally in Ghana, and results on issuing rates are looking very promising.

Several operational challenges were identified by Theiss-Nyland et al. [18] from a four-country Rapid Appraisal Process study including the national and sub-national levels. Within partner organizations, staff felt that ideally ITNs would be managed and distributed by the government, as part of an integrated supply system for health commodities. However, given the resources and capacity currently available, and the experience and expertise brought by partner organizations, both national level government and partner officials stated that, at the current time, separate systems were more functional. Staff expressed concern that there was not enough funding available for the training that was planned and needed. NMCP staff and partner organizations reported limiting ITN trainings to new or inexperienced staff to deal with limited funding and high turnover.

A multi-country review by Willey et al. [19] of 20 African studies of many delivery strategies including ANC clinics and campaigns found that costs were largely comparable across strategies; ITNs were the main cost. Cost-effectiveness estimates were most sensitive to the assumed net lifespan and leakage. Common barriers to delivery included cost, stockouts, and poor logistics. Common facilitators were staff training and supervision, cooperation across departments or ministries, and stakeholder involvement.

A qualitative review in Ghana of sulfadoxine-pyrimethamine (SP) and ITN delivery though ANC clinics showed that stockouts due to procurement delays at the national level [20] resulted in missed opportunities to deliver SP and ITNs to eligible pregnant women at the ANC clinics.

Governments of malaria endemic countries contributed almost a third of total funding in 2020, with investments nearing US$ 1.1 billion. Of this amount, an estimated US$ 0.3 billion was spent on malaria case management in the public sector and more than US$ 0.7 billion on other malaria control activities [1]. Ensuring supply of ITNs through routine channels could be a particularly effective use of these domestic resources.

Theiss- Nyland et al. [21] reported that that greater attention to data collection and use, by both the global malaria community and the integrated programmes, can improve the strength and effectiveness of these distribution channels.

In countries not already doing so, it is recommended that routine CD data be reviewed at least quarterly by the NMCP and relevant partners (e.g., those in the EPI Programme) and that annual data can be collated by the WHO for the WMR. The forthcoming country CD tracker, which will obtain data on all CD activities in country and is under development by the Alliance for Malaria Prevention (AMP), should bring greater focus to and potentially additional resources for CD activities.

### Improving but not yet reaching full potential

Despite extensive operational experience in implementing continuous ITN distribution across many countries, significant challenges remain in reaching intended net users, ensuring consistent ITN supply, and tracking ITNs delivered. While numerous evaluations of ITN mass campaigns have been conducted, relatively few CD implementation systems have been evaluated.

In addition, while significant international and country resources have been invested in developing extensive evidence-based guidance and tools and providing specialized technical assistance to ensure the success of ITN campaigns since 2002, relatively fewer resources have been dedicated to leveraging global partner interest and resources for dedicated support to elevate ITN CD to a similar level to support the maintenance of ITN coverage between campaigns or indeed to replace mass campaigns.

However, the PMI-funded VectorWorks and VectorLink projects have already catalogued global best practices and tested expansion of ITN CD through community and school-based interventions [17].

The “Insights” article 2019 [22] from PMI VectorWorks reiterates that ANC and EPI net distribution save the most lives and must be prioritized as the cornerstone of any country’s distribution strategy, in addition to recurrent mass, school, or community distribution. They point out that, although most countries already include ANC and EPI in their strategy, they have difficulty maintaining consistent supply chains. All too often, ITNs are taken from ANC and EPI to fill gaps in mass campaigns [23]. Several of the discussions with the NMCPs mentioned the issue of rerouting routine nets into mass campaigns.

The global malaria community of practice is turning increasingly towards CD to move away from the arduous and time-consuming effects of mass campaigns. In 2020, to bring greater attention to and increase promotion of CD activities, the AMP re-established the CD Working Group which had been dormant for several years and had previously operated through the RBM Vector Control Working Group.

The PMI VectorLink project has been assisting national programmes to focus on CD and to set up national CD Working groups. Zambia was the first country to do this in 2021 when the National Malaria Elimination Programme established the CD Task Team (Ndoluvu, pers comm).

A recent paper by Haenssgen et al. [24] provides evidence that well-functioning health systems can benefit from mass campaigns but challenges the established wisdom to intensify mass campaigns in weaker health systems to bypass service provision bottlenecks. They point out that mass campaigns do not inherently benefit or damage a health system, but frequent campaigns in weak health system contexts can impede service provision, and they call for an additional burden of proof and active efforts to integrate the services delivered through mass campaigns into routine health services by harmonizing implementation plans and service delivery in weak health system contexts.

Routine health facility–based distribution to pregnant women through ANC and to children at EPI visits should be the prioritized channels for a routine CD approach to ensure that biologically vulnerable populations always have access to ITNs. Some countries have expressed interest in providing nets to women after delivery and to children during outpatient visits. If children are being missed at EPI due to vaccine campaigns, then, if feasible, programmes could consider providing nets during these campaigns.

Other complementary options must be added, as needed, because the ANC and EPI distribution channels alone are, of course, not able to reach older people or those without children and they cannot sustain universal access without mass campaigns or increasing CD activities through community or school-based channels.

CDAs, NMCP discussions, and HMIS ITN data can provide insight into the performance of ITN distribution within these programmes. There is no doubt that greater attention to data collection and use, by both the global malaria community and the integrated programmes, can improve these distribution channels’ strength and effectiveness.

In 2014, Hill et al. stated that “going forward, national malaria programmes and donors alike will have to make difficult decisions to balance costs with the benefits and impact of investments in ITNs. Where choices must be made, high-risk groups (pregnant women and children under five years of age) should be prioritized for the same reason these groups were targeted under the pre-universal coverage WHO strategy” [23].

Encouraging findings are that, overall, issuing rates seem to be improving if we compare the average reported availability rates (number of ITNs reportedly distributed at the national level/number of women reported attending ANC at least once) of 55% for ANC and 34% for EPI respectively [12] with the 65% and 69% rates derived from data from the issuing data from seven countries reviewed in this paper.

The importance of the ANC and EPI routine channels reaching their full potential cannot be underscored enough. These findings of variable results across and within countries, of consistent reasons for variability, and that, although issuing rates seem to be improving, many countries have not yet reached their potential will enable country stakeholders to better understand the contextualized successes and issues within their health system to strengthen routine distribution of ITNs. They also enable global and national programmes to identify key aspects of routine distribution of ITNs for increased focus and resourcing. Although there are encouraging signs of improvement in the issuing rates and some countries are reaching optimal rates, there is still a long way to go until every pregnant woman and young child in sub-Saharan Africa receives the ITN to which they are entitled.

## Supplementary Information

**Additional file 1: Table S1.** Components of ITN CD and key findings and recommendations from PMI VectorLink CDAs

## Data Availability

The datasets used and/or analysed during the current study are available from the corresponding author on reasonable request.

## Abbreviations

AMP: Alliance for Malaria Prevention
ANC: Antenatal Care
CD: Continuous Distribution
CDA: Continuous Distribution Assessment
DHIS2: District Health Information Software
EPI: Expanded Programme for Immunization
HMIS: Health Management Information System
ITN: Insecticide-Treated Net
KINET: Ifakara and Swiss Tropical and Public Health Institute Kilombero Net Project
LMD: Last-Mile Delivery
MR: Measles-Mumps-Rubella
MOH: Ministry of Health
NMCP: National Malaria Control Programme
PMI: United States President’s Malaria Initiative
PSI: Population Services International
RBM: Roll Back Malaria Partnership to End Malaria
SBC: Social Behaviour Change
SMITN: Social Marketing of ITNs Project
SP: Sulfadoxine-pyrimethamine
WHO: World Health Organization
WMR: World Malaria Report
