# Supplementary material for: Multi-country review of ITN routine distribution data: are ANC and EPI channels achieving their potential?
Source: Malar J. 2022 Dec 3;21:366. doi: 10.1186/s12936-022-04373-6 (PMC9719175; doi:10.1186/s12936-022-04373-6)
Supplement: Supplementary file 1 — Additional file 1: Table S1. Components of ITN CD and key findings and recommendations from PMI VectorLink CDAs [file 12936_2022_4373_MOESM1_ESM.docx]

Additional File 1: Table 4: Components of ITN CD and key findings and recommendations from PMI VectorLink CDAs

| Key Findings | Key Recommendations |
| --- | --- |
| Planning and Coordination | |
| Mechanisms were in place with high level MOH support but there was less communication between departments | Improve communication across MOH (EPI/reproductive and child health clinic) NMCP to consider convening a CD Taskforce |
| Lack of CD guidelines or out-dated guidelines | Develop or update distribution guidelines and develop job aides |
| Client Identification | |
| Limited mechanism for identifying the clients | Improve mechanisms and strengthen the capacity of health providers for identifying clients |
| Limited outreach strategies | Explore how to reach clients through outreach services, assisted delivery, schools, community-based distribution etc |
| Quantification | |
| Quantification not meeting the needs due to the centralized quantification exercises | Involve peripheral levels in the quantification process |
| Obsolete quantification tools | Update annually (if not already done) using demography data and other quantification tools or use more accurate health facility population data sources |
| Transport and Stock Management | |
| Lack of transport from district to health facilities identified as one of the leading causes of stockouts | Consider adding vehicles for transportation during budgeting exercises or Last Mile Delivery (LMD) |
| Hard to reach health facilities not supplied | Consider implementing LMD or other options |
| Stock Management tools not available or used inconsistently | Ensure tools are available and staff are trained |
| Frequent stockout at peripheral levels | Need to install a minimal stock alert for supply |
| ITN data not reviewed uniformly | Implement data validation meetings to reconcile malaria data, including ITN stock, and distribution data at all levels |
| ITN Distribution | |
| ITN supply entirely dependent on donor support leading to shortages | Governments should consider self-financing or providing buffer stocks to cover demand if donor stocks run out |
| ITNs not always issued at EPI or at ANC first visit | Share operational guidance with health facilities to support their understanding and respect of distribution instructions and ensure regular monitoring of the implementation of ITNs distribution at the issuing point |
| Personnel and Capacity Strengthening | |
| High turn-over of health facility staff leads to limited knowledge of roles and responsibilities | Include roles and responsibilities for effective management of ITNs in national CD ITN guidelines; consider implementing peer mentorship activities an effective coaching system for new providers |
| Work overload on health care providers | Reorganise work to establish a balanced distribution of tasks and responsibilities |
| Training | |
| Limited training and refresher activities | Use opportunities such as data validation meetings and others to build capacity |
| Outdated training tools and materials | Update training tools and materials |
| Supervision | |
| Checklists not available and/or not appropriate | Review and update supervision checklists to ensure consistent inclusion of ITN distribution, stock, and data management tools |
| Data Management | |
| Sub-optimal data collection and quality control; inaccurate and untimely data ITN re-supply requests from health facilities leads to stockouts. | Capture, control and analyse data and information meticulously for better use. n |
| Non-existent consolidated data collection tools | Consolidate training tools to reduce overload |
| Outdated data collection tools | Ensure tools are up to date, and digitalize if appropriate |
| Communication | |
| Limited SBC budget and communication strategy | Ensure SBC is funded.  Reinforce coordination and develop strategic ITN use messages and messages to reinforce eligibility criteria and that clients can ask for nets. Ensure ANC and EPI providers and community health workers are trained in SBC appropriately. |
| Limited monitoring of the impact of SBC activities on populations to allow for appropriate updates | Regularly monitor the effectiveness of SBC messages to make appropriate and necessary updates |
